# Optimal Waist-to-Height Ratio Values for Cardiometabolic Risk Screening in an Ethnically Diverse Sample of South African Urban and Rural School Boys and Girls

**DOI:** 10.1371/journal.pone.0071133

**Published:** 2013-08-13

**Authors:** Tandi E. Matsha, Andre-Pascal Kengne, Yandiswa Y. Yako, Gloudina M. Hon, Mogamat S. Hassan, Rajiv T. Erasmus

**Affiliations:** 1 Department of Biomedical Technology, Faculty of Health and Wellness Sciences, Cape Peninsula University of Technology, Cape Town, South Africa; 2 NCRP for Cardiovascular and Metabolic Diseases, South African Medical Research Council and University of Cape Town, Cape Town, South Africa; 3 Division of Chemical Pathology, Faculty of Health Sciences, University of Stellenbosch, Cape Town, South Africa; Innsbruck Medical University, Austria

## Abstract

**Background:**

The proposed waist-to-height ratio (WHtR) cut-off of 0.5 is less optimal for cardiometabolic risk screening in children in many settings. The purpose of this study was to determine the optimal WHtR for children from South Africa, and investigate variations by gender, ethnicity and residence in the achieved value.

**Methods:**

Metabolic syndrome (MetS) components were measured in 1272 randomly selected learners, aged 10–16 years, comprising of 446 black Africans, 696 mixed-ancestry and 130 Caucasians. The Youden’s index and the closest-top-left (CTL) point approaches were used to derive WHtR cut-offs for diagnosing any two MetS components, excluding the waist circumference.

**Results:**

The two approaches yielded similar cut-off in girls, 0.465 (sensitivity 50.0, specificity 69.5), but two different values in boys, 0.455 (42.9, 88.4) and 0.425 (60.3, 67.7) based on the Youden’s index and the CTL point, respectively. Furthermore, WHtR cut-off values derived differed substantially amongst the regions and ethnic groups investigated, whereby the highest cut-off was observed in semi-rural and white children, respectively, Youden’s index0.505 (31.6, 87.1) and CTL point 0.475 (44.4, 75.9).

**Conclusion:**

The WHtR cut-off of 0.5 is less accurate for screening cardiovascular risk in South African children. The optimal value in this setting is likely gender and ethnicity-specific and sensitive to urbanization.

## Introduction

The global burden of higher-than-optimal weight is both significant and rising, with most of the increase reported over the last decade. While attention has previously been focused on under-nutrition in African children, recent survey data suggest that overweight rates in male adolescents have increased from 6.3% in 2002 to 11.0% in 2008 and from 24.3% to 29.0% in female adolescents [1]. Similar to adults, obesity is a health concern in children and adolescents as it persists into adulthood with 80% of obese children likely to become obese adults [2]. In some cases many complications associated with obesity are already evident in childhood, necessitating commitment to lifelong treatments at an earlier age. We have previously reported a high prevalence of metabolic syndrome (MetS) in obese and overweight children from South Africa [3]. Central obesity and insulin resistance are dominant features of MetS. As a result, the measurement of waist circumference (WC) has been adopted as criterion for diagnosing MetS by different organizations such as the International Diabetes Federation (IDF) [4] and the National Cholesterol Education Program-Adult Treatment Program (NCEP-ATP III) [5]. The waist-to-height ratio (WHtR) has been proposed as an alternative to waist circumference for the assessment of central obesity when defining the metabolic syndrome (MetS) [6], [7]. The suggestion that WHtR cut-off may be similar in men and women makes it attractive for the quantification of central obesity in children which otherwise, could be very complex when using age-sex-race specific charts. A WHtR cut-off of 0.5 has been proposed for predicting cardiovascular risk [8], and its accuracy has been reported in several studies [9], [10], [11]. Despite its reported advantages and the ease of computing the WHtR, a suitable WHtR cut-off for populations from Africa has yet to be determined. Therefore, in the present study we aimed to determine the WHtR cut-off for children from South Africa, using the presence of at least 2 components of MetS other than WC as an outcome.

## Materials and Methods

### Study Setting and Population

The study setting has been previously described [3]. Briefly, 1272 out of 1960 (65% response rate) learners aged 10–16 years were recruited randomly and proportionally from public or government funded primary and secondary schools, using a list of schools obtained from the Western Cape Education Department between January 2007 and March 2008. Participants with a history of diabetes and learners from private schools were excluded as private schools represented less than 2% of the total number of schools. The study was approved by the Cape Peninsula University of Technology Faculty of Health and Wellness Sciences ethics committee, and the study was conducted according to the Code of Ethics of the World Medical Association (Declaration of Helsinki). Permission to conduct the study was also obtained from the Western Cape Department of Education, school governing bodies and school principals. Written informed consent from parents and oral assent from students was obtained after all the procedures had been fully explained.

### Clinical Measurements

Qualified healthcare professionals performed all the clinical examinations. Blood pressure measurements followed the WHO guidelines [12], and were performed using a semi-automatic digital blood pressure monitor (Rossmax PA, USA) on the right arm in a sitting position. After a 10 minute rest period, three readings were taken at 5 minutes interval and the lowest of the three readings was used in the current analyses. Weight, to the nearest 0.1 kilogram was determined with the subject in light clothing and without shoes and socks, using a Sunbeam EB710 digital bathroom scale, which was calibrated and standardized using a weight of known mass. Height, to the nearest 0.1 centimeters was recorded using a stadiometer with subjects standing on a flat surface at a right angle to the vertical board of the stadiometer. Waist circumference was measured using a non-elastic tape at the level of the narrowest part of the torso as seen from the anterior view. The hip circumference was also measured using a non-elastic tape around the widest portion of the buttocks. All anthropometric measurements were performed three times and the average used for analysis.

### Blood Sample Collection and Analysis

Finger prick blood was used for the estimation of glucose and lipid levels using respectively, the Accutrend GCT glucometer and CardioCheckTM P.A analyzer (Polymer Technology Systems, Inc. USA). The commercial glucometer used in this study had a mean imprecision of <5%, with a range of 1.1–33.3 mmol/L on capillary whole blood.

### Definitions and Calculations

The International Diabetes Federation diagnostic criteria for ages 10 to 16 years old provided by Zimmet et al [4], was used to define metabolic syndrome. Body mass index (BMI) was calculated as weight per square metre (kg/m2) and waist-hip-ratio (WHR) as waist/hip circumferences (cm). The waist-to-height ratio (WHtR) was calculated as waist/height (cm) and the A Body Shape Index (ABSI) [13] derived from the formulae Waist circumference/BMI^2/3^ *height^1/2^. Overweight and obesity status were assessed using age-gender-specific cut-off points international references provided by the International Task Force as developed by Cole and co-workers [14].

### Statistical Analysis

**Figure 1:**
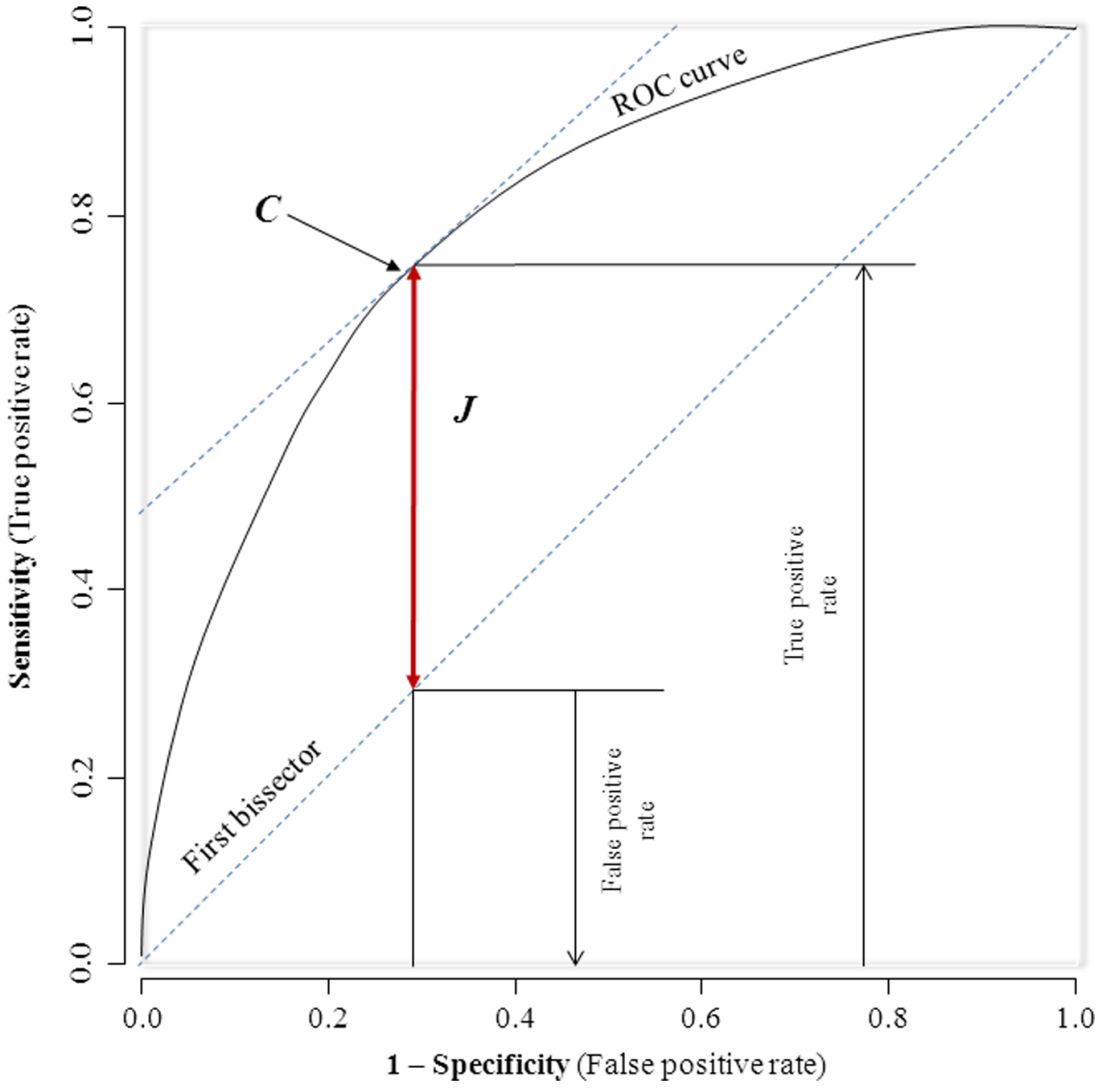
Hypothetical receiver-operating characteristic depicting the Youden index (J) and the optimal cut-point (C).

General characteristics of the study groups are summarized as count and percentage for qualitative variables, mean and standard deviation (SD) for quantitative variables. Group comparisons used chi square tests and equivalents for qualitative variables, and Stutent’s t-test and analysis of the variance (ANOVA) for quantitative variables, with adjustments where relevant through logistic and linear regression models. The *pROC* package [15] of the R statistical software version 2.13.0 [13-04-2011], (The R Foundation for Statistical Computing, Vienna, Austria) was used for receiver operating characteristics (ROC) analyses. The area under the curve (AUC) was then used to assess and compare the ability of waist-to-height ratio and other anthropometric variables to predict the presence of any two components of metabolic syndrome with AUC comparisons through non-parametric methods [16]. The optimal WHtR was determined by applying both the Youden’s index approach [17] and the closest top left point approach [18]. The Youden’s index (J) is estimated as J = sensitivity+specificity –1. Maximizing this index allows to find, from the ROC curve, an optimal cut-off point independently from the prevalence. On the ROC plot (Figure 1), J represents the vertical distance between the ROC curve and the first bisector (chance line or diagonal line through 45°). In the ROC curve the true positive rate is plotted in function of the false positive rate for different cut-off points. Each point on the ROC curve represents a sensitivity/specificity pair corresponding to a particular decision threshold. A test with perfect discrimination has a ROC curve that passes through the upper left corner (100% sensitivity, 100% specificity). Therefore the closer the ROC curve is to the upper left corner, the higher the overall accuracy of the test.

## Results

### Characteristics of the Cohorts

The baseline profile of the participants stratified by race and gender has been presented elsewhere [3]. Herein, the baseline characteristics by gender are presented in Table 1. Girls had higher body mass index, waist and hip circumferences, total-, LDL- and HDL-cholesterol and diastolic blood pressure. However, with the exception of total cholesterol (p = 0.009), age (p = 0.008) and waist circumference (p = 0.045), there were no differential effects of region on baseline characteristics across the genders (all region*gender interaction≥0.05; Table 1).

**Table 1:** General characteristics of participants overall and by gender.

Variables	Boys	Girls	p	Overall	P region	P region*gender
N	496	776		1272		
Age (years)	13.3 (1.9)	13.1 (1.8)	0.106	13.1 (1.8)	<0.0001	0.008
Fasting blood glucose (mmol/L)	4.1 (0.8)	4.0 (0.8)	0.216	4.0 (0.8)	<0.0001	0.243
Triglycerides (mmol/L)	0.77(0.47)	0.82 (0.58)	0.100	0.80 (0.50)	0.0003	0.124
LDL-cholesterol (mmol/L)	2.2 (0.7)	2.3 (0.8)	0.030	2.3 (0.7)	0.0002	0.725
HDL-cholesterol (mmol/L)	1.0 (0.4)	1.1 (0.4)	<0.0001	1.1 (0.4)	<0.0001	0.062
Total cholesterol (mmol/L)	3.4 (0.8)	3.7 (0.9)	<0.0001	3.6 (0.9)	<0.0001	0.009
Systolic blood pressure (mmHg)	107.4 (14.2)	106.6 (12.7)	0.302	106.9 (13.3)	0.003	0.635
Diastolic blood pressure (mmHg)	64.7 (10.6)	65.9 (9.7)	0.048	65.5 (10.1)	0.002	0.707
Height (m)	1.57 (0.14)	1.53 (0.09)	<0.0001	1.54 (0.11)	<0.0001	0.143
Weight (Kg)	48.7 (15.1)	50.0 (13.9)	0.119	49.2 (14.4)	0.003	0.153
Body mass index (kg/m2)	19.4 (3.9)	21.1 (4.6)	<0.0001	20.5 (4.4)	0.133	0.079
Hip circumference (cm)	84.5 (11.1)	88.5 (11.7)	0.0001	86.9 (11.7)	<0.0001	0.110
Waist circumference (cm)	66.6 (9.8)	69.1 (10.4)	<0.0001	68.2 (10.2)	0.011	0.045
Waist-to-hip ratio	0.79 (0.05)	0.78 (0.06)	<0.0001	0.78 (0.06)	<0.0001	0.148
Status for obesity			<0.0001		0.626	0.009
Normal weight, n (%)	428 (86.3)	556 (71.6) (76.6)		984 (77.4) (77.4)		
Overweight, n (%)	46 (9.3)	151 (19.5)		197 (15.5)		
Obese, n (%)	22 (4.4)	69 (8.9)		91 (7.2)		
Region			0.219			NA
Missing, n (%)	21 (4.2)	34 (4.4)		55 (4.3)		
Rural, n (%)	41 (8.3)	71 (9.1)		112 (8.8)		
Semi-urban, n (%)	158 (31.9)	205 (26.4)		363 (28.5)		
Urban, n (%)	276 (55.6)	466 (60.1)		742 (58.3)		
Race			0.121		<0.0001	0.275
Black, n (%)	158 (31.9)	288 (37.1)		446 (35.1)		
Colored, n (%)	281 (56.6)	415 (53.5)		696 (54.7)		
White, n (%)	57 (11.5)	73 (9.4)		130 (10.2)		
Metabolic syndrome (IDF definition)						
High waist circumference, n (%)	36 (7.3)	93 (12.0)	0.006	129 (10.1)	0.897	0.087
High blood pressure, n (%)	40 (8.1)	45 (5.8)	0.114	85 (6.7)	0.011	0.064
High fasting blood glucose, n (%)	27 (5.4)	27 (3.5)	0.090	54 (4.2)	<0.0001	0.262
High triglycerides, n (%)	21 (4.2)	31 (4.0)	0.834	52 (4.1)	0.012	0.249
Low HDL-cholesterol, n (%)	304 (61.3)	343 (44.2)	<0.0001	647 (50.9)	0.0001	0.246
3 components or more, n (%)	17 (3.4)	7 (0.9)	0.001	24 (1.9)	>0.999	0.488
Any 2 components excluding waist circumference, n (%)	63 (12.7)	42 (5.4)	<0.0001	105 (8.3)	0.201	0.123

IDF, International Diabetes Federation.

### Discriminatory Power of Anthropometric variables for Metabolic Syndrome

The prevalence of metabolic syndrome and components based on the IDF definition is described in Table 1. Metabolic syndrome was more frequent in boys than in girls (p<0.001). Low HDL-cholesterol levels were more frequent in both girls and boys followed by high waist circumference in girls, whilst in boys the blood pressure was the second common component. The discriminatory power of anthropometric variables for the prediction of any two IDF-defined component of metabolic syndrome (exclusive of waist circumference) is shown in Table 2. The highest point estimate of AUC when comparing the four clinical measures of adiposity was recorded for waist circumference in boys and the overall sample, respectively 0.740 and 0.681, but in girls it was the hip circumference, 0.647. However, no significant differences were apparent when comparing WC with hip circumference (overall p = 0.158, boys p = 0.540, girls p = 0.824). Although point estimates of the AUC for the waist-to-height-ratio were always the fourth (overall 0.619, boys 0.678, girls 0.618), they showed significant differences only when compared to BMI overall (p = 0.035) and waist circumference overall (p = 0.0003) and in boys (p = 0.018), but not in girls (p = 0.268), nor when compared with other variables (Table 2).

**Table 2:** Adipometric variables and discrimination of any two components of metabolic syndrome in the derivation sample.

Variables	AUC (95% CI)	p-value for differences in AUC				
		vs. BMI	vs. WC	vs. Hip	vs. WHR	vs. WHtR
**Overall**						
BMI	0.654 (0.599–0.708)	–				
WC	0.681 (0.628–0.733)	0.013	–			
Hip	0.661 (0.611–0.711)	0.589	0.158	–		
WHR	0.594 (0.534–0.655)	0.075	0.002	0.080	–	
WHtR	0.619 (0.562–0.677)	0.035	0.0003	0.070	0.352	–
ABSI	0.503 (0.443–0.563)	<0.0001	<0.0001	<0.0001	0.096	0.010
**Boys**						
BMI	0.726 (0.655–0.796)	–				
WC	0.740 (0.669–0.811)	0.232	–			
Hip	0.731 (0.664–0.797)	0.771	0.540	–		
WHR	0.617 (0.535–0.698)	0.009	0.001	0.016	–	
WHtR	0.678 (0.600–0.757)	0.059	0.018	0.134	0.044	–
ABSI	0.505 (0.422–0.587)	<0.0001	<0.0001	<0.0001	0.0003	0.0003
**Girls**						
BMI	0.631 (0.543–0.719)	–				
WC	0.642 (0.564–0.719)	0.579	–			
Hip	0.647 (0.571–0.724)	0.517	0.824	–		
WHR	0.473 (0.376–0.570)	0.039	0.033	0.006	–	
WHtR	0.618 (0.532–0.704)	0.604	0.268	0.433	0.086	–
ABSI	0.527 (0.434–0.620)	0.021	0.040	0.008	0.296	0.144

ABSI, A Body Shape Index; AUC, area under the receiver-operating characteristic curve; BMI, body mass index; Hip, hip circumference; WC, waist circumference; WHR, waist-to-hip ratio; WHtR, waist-to-height ratio; 95% CI, 95% confidence interval.

### Optimal Waist-to-height Ratio Cut-off Values

The two approaches used to derive the optimal cut-off values identified a similar cut-off in boys and girls but two different values in the overall sample (Table 3). The optimal waist-to-height ratio value for diagnosing any two components of metabolic syndrome based on the Youden’s index method was 0.465 (sensitivity 41.9%, specificity 77.2%) in the overall sample, 0.455 (42.9%, 88.4%) in boys and 0.465 (50.0%, 69.5%) in girls. Equivalents results based on the closest-top left point approach were 0.435 (56.2%, 59.5%) in the overall sample. Different thresholds were also observed when deriving the optimal cut-off values in different regions and race whereby the highest cut-off were observed in semi-rural (0.505) and white children (0.475).

**Table 3:** Optimal waist-height cut-off values and measures of performance in the derivation sample.

WHtR thresholds	Methods	Sensitivity	Specificity	PPV	NPV	Youden index
**Overall**						
0.465	Youden index	0.419	0.772	0.142	0.937	0.191
0.435	CTL	0.562	0.595	0.111	0.938	0.157
0.500		0.305	0.858	0.162	0.932	0.163
**Boys**						
0.455	Youden index	0.429	0.884	0.351	0.914	0.313
0.425	CTL	0.603	0.677	0.213	0.921	0.280
0.500		0.254	0.940	0.381	0.896	0.194
**Girls**						
0.465	Youden index	0.500	0.695	0.086	0.96	0.195
0.465	CTL	0.500	0.695	0.086	0.96	0.195
0.500		0.381	0.809	0.103	0.958	0.190
**Rural**						
0.415	Youden index	0.818	0.396	0.129	0.952	0.214
0.415	CTL	0.818	0.396	0.129	0.952	0.214
0.500		0.182	0.851	0.118	0.905	0.033
**Semi-rural**						
0.505	Youden index	0.316	0.871	0.222	0.916	0.187
0.455	CTL	0.447	0.711	0.153	0.917	0.158
0.500		0.316	0.849	0.197	0.914	0.165
**Urban**						
0.475	Youden index	0.451	0.808	0.146	0.952	0.259
0.435	CTL	0.647	0.593	0.105	0.958	0.240
0.500		0.333	0.862	0.152	0.946	0.195
**Black**						
0.465	Youden index	0.500	0.754	0.120	0.957	0.254
0455	CTL	0.536	0.699	0.107	0.957	0.235
0.500		0.357	0.832	0.125	0.951	0.189
**Coloured**						
0.425	Youden index	0.644	0.563	0.121	0.944	0.207
0.425	CTL	0.644	0.563	0.121	0.944	0.207
0.500		0.288	0.877	0.179	0.930	0.165
**White**						
0.475	Youden index	0.444	0.759	0.229	0.895	0.203
0.445	CTL	0.556	0.634	0.196	0.899	0.190
0.500		0.278	0.839	0.217	0.878	0.117

CTL, closest top left; NPV, negative predictive value; PPV, positive predictive value; WHtR, waist-to-height ratio.

## Discussion

The results from this study do not support the use of a universal cut-off level for South African children across various ethnic and gender groups. The two approaches used to derive cut-offs yielded similar cut-off in girls, and the value was higher in girls compared with boys. Moreover, derived WHtR cut-off values differed substantially amongst the regions and ethnic groups investigated, whereby the highest cut-off was observed in semi-rural and white children. Our results also show that the WHtR achieved the least point estimates of AUC than the four measures of adiposity in discriminating the presence of any two components of metabolic syndrome (exclusive of waist circumference).

The correction of waist circumference to height has been conceived to avert the need for age-sex-ethnic charts in the measurements of central obesity in children. Though a single cut-off of 0.5 has been proposed for both genders and many ethnic groups [6], [8], different cut-off values have also been reported. For example, in Chinese children, 0.445 and 0.485 have been suggested as optimal cut-offs for overweight and obesity, respectively [19], whilst in Australian children the most suitable cut-off values were 0.46 in boys and 0.45 in girls for overweight and 0.48 in male subjects and 0.47 in female subjects for obesity [20]. Although some investigators have raised concerns about the statistical validity of WHtR [21], [22], a recent report has demonstrated that the predictive ability of WHtR is not improved by age and sex-specific exponents to properly adjust for height [23]. It is well documented that a subset of obese individuals are metabolically normal, therefore the notion that the WHtR can accurately estimate visceral adiposity and insulin resistance [24] is equally appealing. Visceral fat (VAT) as opposed to subcutaneous fat is strongly associated with cardiometabolic risk factors in children [25]. In this regard, several reports have provided evidence demonstrating the usefulness of WHtR in the identification of children and adolescents with cardiometabolic risk factors [26], [27], [28], [29]. Likewise, the WHtR cut-offs to identify cardiometabolic risk factors are not uniform. In our data the values with the highest sum of sensitivity and specificity for the identification of at least two components of the MetS were 0.455 in boys and 0.465 in girls based on the Youden index. Furthermore, values obtained also varied when the cohort was stratified by region and race and were always lower than the proposed 0.5 cut-off except in children from the semi-rural region. In Korean children aged 10–15 years the WHtR values corresponding to a derived optimal cut-off for the visceral fat area were 0.54 in boys and 0.61 in girls [30]. Another study in children aged nine to 15 years from schools in the North West province of South Africa reported a value of 0.41 as an optimal WHtR cut-off value to identify children with hypertension, but found WHtR cut-off value of 0.5 to be a better predictor for high blood pressure in the same sample [29]. In contrast the WHtR cut-off of 0.41 to 0.44 was reported as the average value in Japanese children with a normal physique [27]. The discrepancies in these studies including ours is most probably due to the differing outcomes used to derive the threshold and/or the ethnic differences in body fat patterning that are linked to ethnic variability in cardiometabolic risk [31]. Moreover, the anatomical site for the measurement of WC differs in these studies. We measured the WC at the level of the narrowest part of the torso as seen from the anterior view, whilst another study involving South African children measured the WC half way between the superior ridge of the ilium and the lower border of the lowest floating rib [29]. Although WC measurements strongly reflect abdominal adipose tissue, recent data suggests that other sites of measure are more associated with visceral adipose tissue in children [32]. It should be noted that, while the clinical impact of a prognostic index for selecting people at high risk for a disease from those not at risk in a given population, should satisfy several criteria, to maximize the chances of having the prognostic index adopted and applied in the clinical practice, it has also to be easy to use, which included where relevant, adopting cut-points that are easy to remember. Therefore, in settings where the context-specific optimal WHtR cut-off is not appreciably different from the recommended 0.5, a conservative and simple approach may consist of scarifying the precision by sticking to the 0.5. But where differences are likely to result in a reclassification of the risk of a large number of people; context-specific cut-offs should be preferred.

The strength of the present study is our mixed sample that has allowed simultaneous investigation of the research question across gender, ethnic and regional subgroups. Additionally we used two different methodological approaches to derive the cut-offs, based on an outcome that combined at least two other cardiometabolic factors; which many previous studies did not achieve. Limitations include the smaller size of some subgroups investigated and the fact that our sample was not a national representation. Furthermore, the study was limited to public schools primarily located in the lower to middle income areas. Blood tests in our study were based on finger prick sample and point-o-care instruments which may provide less accurate results than usual laboratory methods using venous samples. Although anthropometric measurements were performed three times and the averages used for analysis, measurements were performed by one individual, consequently inter- and intra-operator reproducibility could not be ascertained. Puberty was not accounted for in the statistical analyses as rapid growth in body size accompanied by marked changes in body composition is observed during pubertal stage. Lastly, diet and physical activity status, factors that are known to affect MetS risk profile were not taken into consideration.

In conclusion, our analyses suggest that the ‘one size fits all’ rule regarding the WHtR cut-off for screening cardiovascular risk in children may not be valid in this setting, which in major ways corroborate the findings from other settings. Furthermore WC alone in major ways outperformed WHtR, and always had higher point estimates than other adipometric variables in discriminating the presence of any two components of metabolic syndrome. Taken together, our data suggest that the WC is likely the best performing adipometric variable for the prediction of metabolic syndrome. However, to be able to derive age and gender specific WC cut-offs for application in a given setting, WC growth charts are needed, which are not yet available for South African children. Therefore at least for the time being, WC may not be the preferred adipometric marker in this setting. In the interim, a larger and representative study to determine and validate a WHtR cut-off suitable for African children and adolescents has value.
